# Winning the Blue Sky Defense War: Assessing Air Pollution Prevention and Control Action Based on Synthetic Control Method

**DOI:** 10.3390/ijerph191610211

**Published:** 2022-08-17

**Authors:** Haotian Zhang, Xiumei Sun, Xueyang Wang, Su Yan

**Affiliations:** Business School, Shandong University of Technology, Zibo 255000, China

**Keywords:** air pollution control and prevention action, air pollution, synthetic control method, PM_2.5_

## Abstract

Undoubtedly, the rapid development of urbanization and industrialization in China has led to environmental problems, among which air pollution is particularly prominent. In response, the Chinese government has introduced a series of policies, including the Air Pollution Control and Prevention Action Plan (*APPA*), which is one of the most stringent environmental regulations in history. The scientific evaluation of the implementation of this regulation is important for China to win the battle of blue sky. Therefore, this study uses a synthetic control method to explore the effects of *APPA* on air pollution (*AP*) based on data of 30 provinces from 2000 to 2019. The study concludes that (1) *APPA* significantly reduces *AP* in the treatment provinces, and subsequent robustness tests validate our findings. However, the persistence of the policy effect is short in some provinces, and the rate of *AP* reduction slows down or even rebounds in the later stages of the policy. (2) The reduction effect of *APPA* varies significantly between regions and provinces. (3) The results of mechanism tests show that *APPA* reduces *AP* through high-quality economic development, population agglomeration, control of carbon emissions, and optimization of energy structure. Based on the above findings, targeted recommendations are proposed to promote *AP* control in China and win the blue sky defense war.

## 1. Introduction

China’s rapid economic development is accompanied by massive fossil energy consumption and serious environmental pollution [1]. In recent years, air pollution (*AP*), represented by hazy weather, has attracted widespread public, media, and government attention [2]. In addition to causing a series of related diseases and posing a serious threat to the health of residents, *AP* also causes huge economic losses and hinders the sustainable development of society [3,4]. In the 1970s, air pollution management in developed countries focused mainly on sulfur and nitrogen oxides, and only in the 21st century did they start to shift to managing particulate matter such as PM_2.5_ and PM_10_. Part of these particulate matter comes from the direct emissions of pollutants, such as industrial waste gas, and part of it comes from sulfur and nitrogen oxides. China’s air pollution management started late, and it was not until 2012 that PM_2.5_ was included as an air quality testing standard [5]. According to the 2013 China Environmental Quality Bulletin, only three of the 74 cities that have implemented fine particulate matter (PM_2.5_) monitoring have met China’s National Ambient Air Quality Standards (NAAQS), and more cities are experiencing problems such as exploding *AP* indices and haze [6]. Hourly PM_2.5_ concentrations in Beijing even exceeded 1000 μg/m^3^, which is 40 times higher than the World Health Organization (WHO) health standard level. PM_2.5_ exposure caused about 8.9 million deaths worldwide in 2015, more than a quarter of which occurred in China [7].

**Figure 1:**
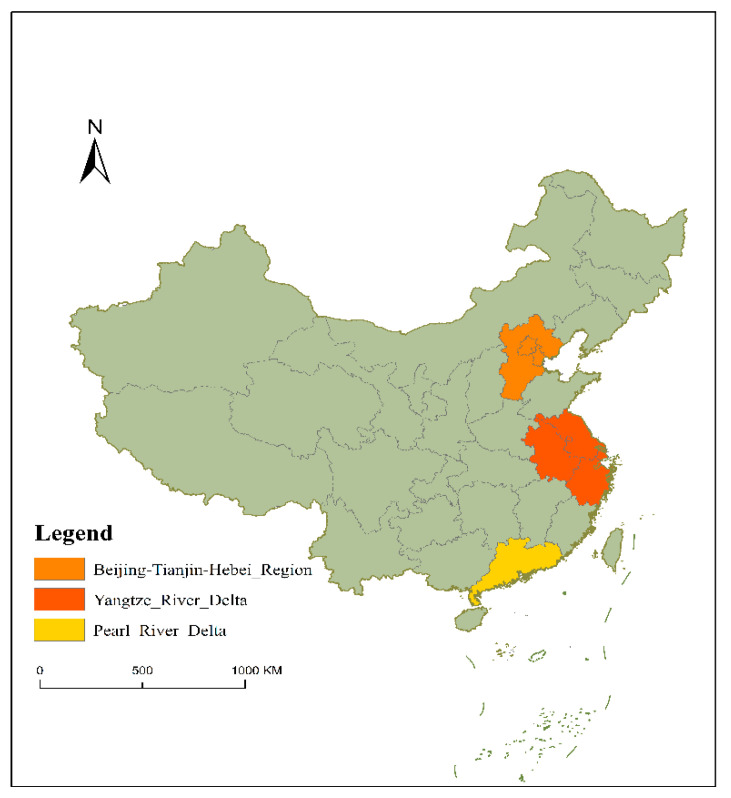
*APPA* implementation focus areas.

In response to China’s growing environmental and *AP* problems, the Chinese government has introduced measures to improve air quality and reduce *AP*. The State Council promulgated the Air Pollution Prevention and Control Action Plan (hereafter referred to as “*APPA*”) in September 2013, known as the most stringent air pollution control system in history [8]. The policy puts forward more specific goals for future *AP* management and sets out the implementation plan for *AP* prevention and control in ten areas. The Beijing–Tianjin–Hebei region, Yangtze River Delta, and Pearl River Delta are facing more serious *AP* problems due to the concentration of resource elements and rapid economic development [9,10,11] (Figure 1). Therefore, one of the important goals of *APPA* is to reduce the concentration of fine particles in the Beijing–Tianjin–Hebei region, Yangtze River Delta, and Pearl River Delta regions to improve air quality.

Has the policy achieved the government’s intended goals? Have the key provinces where the policy was implemented produced significant changes in air quality compared to other provinces? Although some scholars explored the effects of *APPA*, these studies are mostly based on the multiplication method from an overall perspective. The conclusions of these studies may be biased because the assumption of parallel trends in the difference-in-difference method is often not satisfied due to the specificity of the policy implementation focus provinces (e.g., Beijing and Shanghai). Facing the assessment of policy effects with significant differences between samples, it is better to use the synthetic control method to construct a sample similar to the treatment group.

Based on the above motivation, an accurate and objective assessment of the implementation effect of *APPA* is of great practical significance to further promote *AP* control and win the battle for the blue sky. The contributions of this paper are: (1) This study adopts a synthetic control method (SCM) to assess the impact of *APPA* policy on *AP*. Compared with the traditional Difference in Difference (DID) method, it avoids the endogeneity problem brought by excessive subjectivity in sample selection and the bias brought by the treatment and control groups not satisfying parallel trends. It can obtain more specific research conclusions for each province. (2) Comparing previous studies that focused only on the overall effect of the policy, this study focused on examining the regional heterogeneity of policy effects for regional policies, intending to propose more targeted policies for different provinces to promote regional *AP* reduction. (3) The mechanism test of *APPA* is explored to provide paths for improving air quality.

The remainder of this paper is structured as follows. Section 2 presents the literature review and policy background. Section 3 provides the method and data source. Section 4 presents the results and discussion. Section 5 provides the conclusion and policy implications.

## 2. Literature Review and Policy Background

### 2.1. Literature Review

Numerous studies have shown that *AP* significantly threatens human health. Studies by the World Health Organization show that more than 7 million people worldwide die each year from *AP*, and about 90% of the population is exposed to high air concentrations. Long-term exposure to the harsh atmospheric environment increases the risk of respiratory infections, cardiovascular diseases, lung cancer, and other diseases and threatens the population’s lives [12]. On the one hand, *AP* increases mortality and disease, creating a burden of health costs [13]. On the other hand, reduced environmental quality can damage the central nervous system, threaten people’s mental health and increase the risk of suicide [14]. In addition, *AP* can cause serious economic losses by increasing health care expenditures, reducing labor productivity, and impeding human capital mobility [15].

The causes of *AP* are multifaceted. Economic development is undoubtedly one of the most important factors affecting *AP* in a region [16]. Some scholars believe that there is an inverted U-shaped curve between the economy and the environment; i.e., *AP* increases with the level of economic development. However, at higher levels of economic development, *AP* begins to decrease as the level of economic development increases [17]. This phenomenon is similar to the inverted U-curve proposed by Kuznets for income disparity and GDP per capita and is also known as the environmental Kuznets curve [18]. However, some scholars believe that the relationship between economic development and environmental pollution is positive U and N-shaped [19,20]. Similarly, past studies have had no uniform conclusion on the relationship between population and *AP*. Some believe that population concentration promotes industrial clusters and urban scale, which is conducive to the scale effect and improves urban environmental quality [21]. However, others believe that population agglomeration causes more pollution emissions [22,23]. In addition, the excessive burning of fossil fuels produces large amounts of carbon dioxide, contributing to *AP* [24]. Of course, the industrial structure is also a key factor in resolving the conflict between economic development and the environment [25].

Various environmental policies have always been one of the main instruments of the government to combat *AP*, especially in the top–down command-and-control environmental management system in China, where the government is supposed to be the leading force in combating *AP* [26]. Over the past period, many scholars have conducted in-depth analyses of the effects of different *AP* prevention and control policies [27,28]. Of course, the *APPA* has also attracted extensive attention from scholars since its introduction. Many scholars have studied the effects of green total factor productivity [29], business investment [30] and population health [31]. Many scholars have explored the policy effects of *APPA* from the perspectives of green total factor productivity, enterprise investment, and residents’ health. Meanwhile, some scholars have also studied the impact of *APPA* on pollutant emissions. For example, Zhang [32] using the DID method investigated the impact of *APPA* on carbon emissions based in 285 cities. After implementing *APPA*, Yang [33] used the DID method to investigate the major *AP* concentrations in 125 prefecture-level cities. After *APPA* implementation, the above studies generally concluded that *APPA* has contributed to *AP* reduction, but they all focused on the overall level of policy implementation.

In summary, many studies focused on *AP*, and some focused on the effects of *APPA*. However, most studies often choose the multiplication method, which mainly explores the overall perspective of policy implementation. However, in the policy implementation process, each province differs because of factors such as resource endowment, economic development level, and the use of the traditional DID will not satisfy the parallel trend test and thus produce bias. Therefore, this study adopts the synthetic control method to investigate the impact of *APPA* on air quality at the provincial level and then investigate the mechanism on *AP*, which can help propose more localized policy recommendations and provide reference for promoting future *AP* reduction actions in China.

### 2.2. Policy Ground

The Chinese government has issued several policies to improve air quality in light of the increasingly frequent environmental pollution problems. In the Eleventh Five-Year Plan, China proposed to reduce energy consumption per unit of GDP and national sulfur dioxide emissions by 20% and 10%, respectively, in 2010 compared to 2005 [34] in 2010. In the 12th Five-Year Plan, China aims to reduce energy consumption per unit of GDP and national emissions of sulfur dioxide and nitrogen oxides by 16%, 8% and 10%, respectively, by 2015 compared to 2010 [35].

The Air Pollution Control and Prevention Action Plan, released by the State Council in 2013, is a general management plan designed to treat *AP*. Its content mainly covers many aspects such as increasing the comprehensive treatment of pollution sources, adjusting and optimizing the industrial structure, accelerating the green technological transformation of enterprises, increasing the supply of clean energy, strict energy-saving and environmental protection access, establishing a regional integrated management mechanism, establishing a monitoring, early warning and emergency response system and mobilizing all people to participate in environmental protection. The main goal of *APPA* is to reduce the concentration of respirable particulate matter in cities at the prefecture level. Therefore, it is important to investigate the policy effects of *APPA* to improve air quality and win the blue sky defense war in China.

## 3. Method and Materials

### 3.1. Syntenic Control Method (SCM)

Assessing the effect of a policy implemented in a given area is usually not possible by directly comparing the change in outcomes before and after the implementation of the policy due to possible time trends or confounding events. Moreover, the assumption of parallel trends is not always satisfied, so multiplicative difference or fixed effects models are not always appropriate. Abadie provides a good way to construct a counterfactual control group: a synthetic control method [36,37,38]. This approach allows for the selection of optimal weights for linear combinations based on data-driven, avoiding the arbitrariness of the researcher’s subjective choice of control group and avoiding extrapolation bias, and it is well suited for assessing the impact of *APPA* on *AP*.

The sample for this study was selected for 30 provinces (except Tibet). The provinces in the key regions of *APPA* implementation were used as the treatment group—Beijing, Tianjin, Hebei, Anhui, Shanghai, Zhejiang, Jiangsu, and Guangdong—and the other provinces were used as the control group. The other treatment group provinces were excluded from the sample selection process of the control group. The synthetic control method model was constructed as follows.

We assume that K + 1 provinces can be observed in $t\in \left[1,\;T_{0}\right]$ within the *AP* data, assuming that area i (*i* = 1) serves as the treatment group for the *APPA* shock and the other K provinces (*i* > 1) serve as the control group.${AP}_{it}^{N}$ represents the *AP*s without and with *APPA* shocks at time point t. We assume that $\alpha_{1t}=AP^{I}-AP^{N}$, $\alpha_{1t}$ represents the net effect of the policy in *i* province. This study refers to Abadie’s study and extracts the impact factors for areas not affected by the policy (*i* > 1).

(1) $${\mathrm{AP}\;}_{it}^{N}=\theta_{t}Control_{t}+\gamma_{t}\mu_{i}+{year}_{t}+\varepsilon_{it}\;$$

In Equation (1), the $Control_{t}$ is the control variable, the matching variable for SCM, and $\gamma_{t}\mu_{i}$ represents the individual and time interaction effects. In addition, the *K* provinces that do not receive policy effects are assigned weights *w*_2_, *w*_3_, *… w_k+_*_1_ *(w*_2_ *+ w*_3_ *+…+w_k+_*_1_ *=* 1, *w_i_* > 0), which are weighted to obtain Equation (2):


(2) $$\sum_{k=2}^{K+1}w_{k}AP_{kt}^{N}=\theta_{t}\sum_{k=2}^{K+1}w_{k}Control_{k}+\gamma_{t}\sum_{k=2}^{K+1}w_{k}\mu_{k}+\sum year+\sum_{k=2}^{K+1}w_{k}\varepsilon_{kt\;}\;\;$$

To reduce the error of the resultant variables in the treatment and synthesis groups while making the other matching features fit better, we set a constraint that:

(3) $$\{\begin{array}{c} \overset{K+1}{\underset{k=2}{\sum }}w_{k}^{*}AP_{k1}^{N}={AP}_{11}\\ \overset{K+1}{\underset{k=2}{\sum }}w_{k}^{*}{AP}_{k2}^{N}={A\;}_{12}\\ \overset{K+1}{\underset{k=2}{\sum }}w_{k}^{*}\;AP_{kT_{0}}^{N}=AP_{1T_{o}}\\ \overset{K+1}{\underset{k=2}{\sum }}w_{k}^{*}Control_{k}=Control_{1} \end{array}\;$$

(4) $$AP_{1t}^{I}-\sum_{k=2}^{K+1}w_{k}^{*}\;AP_{kt}=\sum_{k=2}^{K+1}w_{k}^{*}\sum_{s=1}^{T_{s}}\lambda_{t}{\left(\sum_{n=1}^{T_{s}}\lambda_{n}^{\prime }\lambda_{n}\right)}^{-1}\lambda_{s}^{\prime }\left(\varepsilon_{js}-\varepsilon_{1s}\right)-\sum_{k=2}^{K+1}w_{k}^{*}\left(\varepsilon_{kt}-\varepsilon_{1t}\right)$$

In Equation (4), if there exists $\sum_{n=1}^{T_{0}}\lambda_{n}^{\prime }\lambda_{n}$, then for any $t⩽T_{0}$, and the right-hand side of the formula tends to 0, then it is possible to use the *K* control group samples in period *t*$\;\left(t⩽T_{0}\right)$. If the *AP* of the treatment group provinces is fitted, then a set of *K* weight matrices of the province values is taken so that the treatment group before the policy is the same as the *AP* of the matched group.

In this paper, we further construct the weight matrix as follows.

(5) $$\{\begin{array}{c} W^{*}=\left(w_{2}^{*},w_{3}^{*},\cdots ,w_{K+1}^{*}\right)\\ \|X_{1}-X_{0}W\|=\sqrt{{\left(X_{1}-X_{0}W\right)}^{\prime }V\left(X_{1}-X_{0}W\right)} \end{array}$$

In Equation (5), *X*_1_ and *X*_0_ represent the matrices of treatment and control group characteristic variables, respectively. *W* is the surrogate weight matrix. *V* is a K-dimensional diagonal matrix with non-negative weights for all diagonal elements, reflecting the relative importance of the corresponding predictor variables for the outcome variable. Obviously, the optimal solution of this constrained minimization problem depends on the diagonal matrix *V*. The mean square error in performing synthetic control estimation is minimized by the choice of *V*. The optimal matrix W* is derived using a reasonable choice of *V* as the matrix composed of the weights of the *K* provinces in the synthetic group.

In summary, the marginal effect of *APPA* on *AP* is


(6) $$\alpha_{1t}=AP_{1t}^{I}-\sum_{k=2}^{K+1}w_{k}^{*}\;AP_{kt}^{N}$$

In Equation (6), the indicator is the treatment effect of the *APPC* shock on the *AP* effect in the treatment group’s province. It is important to note that in the application of Abadie et al. for the synthetic control method, two important criteria are pointed out. First, the difference between the synthetic and treatment groups in matching variables before the occurrence of the exogenous shock event is not significant, while the difference between the two groups’ independent variables is not significant. Second, there is a significant difference between the synthetic and treatment groups in dependent variables after the occurrence of the exogenous shock, and such a difference, after excluding the effect of other matching variable difference characteristics, can be considered to be due to exogenous event shocks [36]. This difference can be attributed to exogenous event shocks after excluding the effect of other matching variables.

This study defines the weights for each province according to the following rules.

(1) Use the eight *APPC* focus provinces before 2013 as the treatment group.
(2) Match control group provinces for each treatment group province with no treatment group provinces included in the matched sample.
(3) Select reasonable weights by synthetic control method to construct synthetic group provinces for treatment group provinces.
(4) Derive the optimal weight matrix using a reasonable choice of the semi-positive definite matrix. Specifically, the average values of each matching index from 2000 to 2012 and the provincial air quality in 2000, 2005, and 2010 are taken. The weight matrix is derived by solving a system of joint cubic equations so that the weighted average of the matching variables of the synthetic group of provinces in each year is comparable to the values taken by the matching variables of the treatment group of provinces, and the weights are greater than 0.
(5) The annual means of matching variables in the synthetic group were calculated and compared with the treatment group, making the matching variables in the 2000–2012 treatment group close to those in the synthetic group.

### 3.2. Variables and Data Source

#### 3.2.1. Dependent Variable

Common indicators for measuring *AP* include SO_2_, NO_2_, and Respirable Particulate Matter (RPM). PM_2.5_ can remain in the atmosphere for longer while being transported over longer distances by air currents, which has a greater impact on the environment and a greater degree of risk to human health than other pollutants [39]. Therefore, this paper selects PM_2.5_ as the most important pollutant. Therefore, the concentration of PM_2.5_ is chosen to measure the *AP* level in this paper.

#### 3.2.2. Predictor Variables

(1) Economic Development (*GDP*): Many studies in the past have demonstrated a strong link between the level of economic development and air quality [40]. In this study, GDP per capita was used to measure the economic development level of the region.
(2) Population Size (*POP*): The population size reflects to some extent the level of industrialization and urbanization of the region, and the population size affects the level of economic development and thus the *AP* in the city [41]. In this study, the year-end population of the region is used to express the population size.
(3) Industrial Structure (*IS*): Differences in regional industrial structure can cause regional differences in pollution, and traditional industries represented by secondary industries are often the main source of pollution in a region [42]. The traditional industry represented by the secondary industry is often the main source of pollution in a region. In this study, the proportion of the output value of the secondary industry is used to reflect the industrial structure.
(4) Carbon Emissions (*CO_2_*): Many studies in the past have proven that carbon dioxide is one of the main sources of atmospheric pollution and that carbon emissions of a region are closely related to its air quality [43]. The carbon emissions are calculated as follows.

(7) $$Carbon_{it}=\sum \begin{array}{c} Energy_{it}\times \eta_{j}\left(i=30;j=1,2,\ldots ,9\right) \end{array}$$

In Equation (7), $Carbon_{it}$ represents the carbon emissions of province *i* in *t* year, while *η_j_* is the carbon emission factor of the *j* energy consumption (Table 1). According to the China Energy Statistical Yearbook caliber, the final energy consumption types are divided into nine categories (Raw Coal, Coke, Crude Oil, Gasoline, Kerosene, Diesel, Fuel oil, Liquefied Petroleum Gas, and Natural Gas). The conversion coefficients of the nine categories of energy are shown in Table 1.

### 3.3. Data

The variables are measured and sourced as shown in Table 2. PM_2.5_ data were obtained from the Atmospheric Composition Analysis Organization. The predictor variable data were obtained from each province’s China Statistical Yearbook and Statistical Yearbooks. For the few missing values, this study used linear interpolation to supplement them. The descriptive statistics of the variables are shown in Table 3. It can be seen that the maximum, minimum and mean values of the annual average PM_2.5_ concentrations of the treatment and control groups are significantly different. In addition, the mean values of annual average PM_2.5_ concentrations are 47.7 μg/m^3^ and 38.52 μg/m^3^, both of which did not meet the national standard of annual average concentration level 2 (35 μg/m^3^). In the subsequent tests, we logged the variables in order to reduce the absolute differences between the data [44].

**Figure 2:**
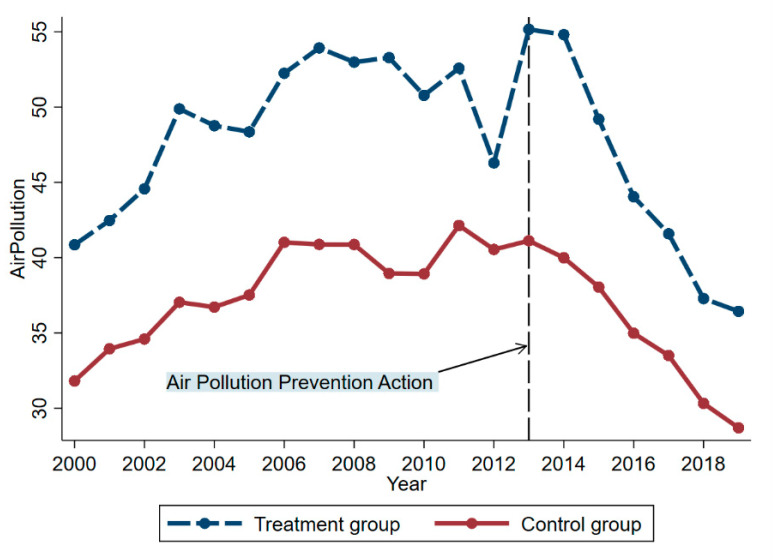
Temporal trends of *AP* in the treatment and control provinces.

Figure 2 shows the time trends of *AP* in the treatment and control provinces. It can be seen that the *AP* levels in the treatment provinces are significantly higher than those in the control provinces, and the *AP* levels in the treatment provinces do show a significant downward trend after 2013. We will further verify whether *APPA* causes this downward trend in the later experiments and verify the changes of *AP* in each treatment province from a microscopic perspective.

## 4. Results and Discussion

### 4.1. Variable Matching

Table 4 shows the control of predictor variables for the synthetic control method. It can be seen that most of the predictor variables for the provinces in the focus group of policy implementation and their synthetic provinces do not have significant deviations, indicating that the predictor variables do not differ significantly between the treated and synthetic provinces. It should be noted that the Tianjin sample was excluded because the *AP* in Tianjin was significantly higher than that in other provinces, and no suitable province could be selected to match it. After the matching process, the differences in the pre-predictor *AP* between the treatment group provinces and the synthetic provinces were also not significant. The above matching results indicate that the characteristics of the treatment and control groups are similar and can be subjected to synthetic control analysis.

Table 5 shows the selection and weights of the synthesized provinces. For example, the provinces involved in synthesizing Beijing are Henan, Hubei, Ningxia, and Shandong, where the largest weight is Henan (0.565), indicating a higher similarity between Henan and Beijing; the lowest weight is Ningxia (0.044), indicating a lower similarity between Ningxia and Beijing.

### 4.2. Baseline Results

**Figure 3:**
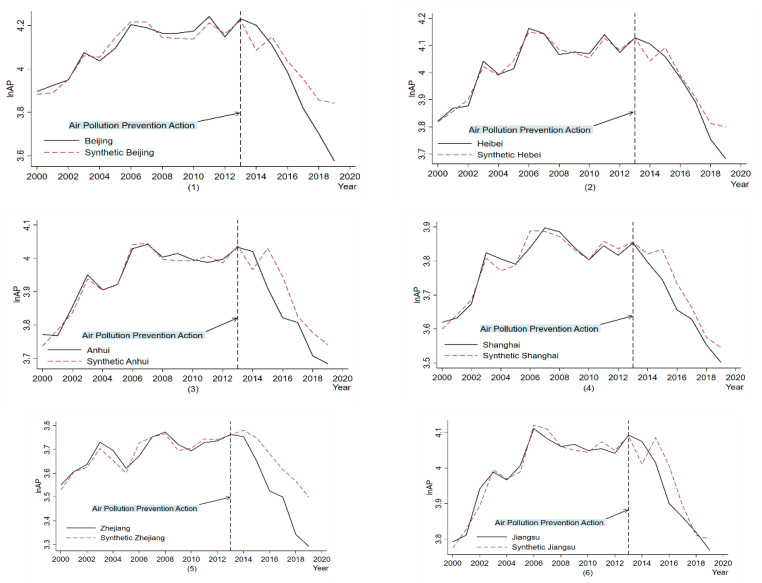

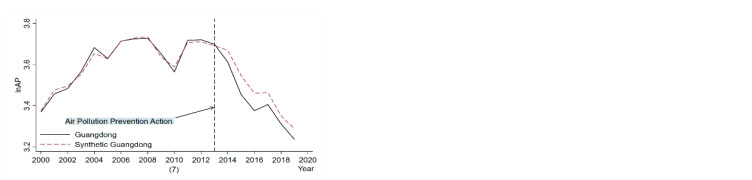
Air pollution in the treatment and synthetic provinces.

We analyze the trend of *AP* changes in the treatment and synthetic provinces, and Figure 3 shows the *AP* changes after *APPA*. The solid black line represents the *AP* level in the treatment province, the red dashed line represents the *AP* level in the synthetic province, and the black dashed line represents the policy implementation time, i.e., 2013.

Before the implementation of *APPA*, the synthetic and treatment provinces generally fit well, such as Guangdong and Hebei, which showed excellent fitting trends. Beijing and Shanghai provinces still have a good overall fit, although there are individual years with fit deviations before *APPA*.

After the implementation of *APPA*, the *AP* in the treatment provinces showed a more significant decrease compared to the synthetic provinces. It indicates that the implementation of *APPA* did promote the reduction in *AP* in the treatment provinces. At the same time, we also found several interesting phenomena: (1) The policy effect in some provinces is only more effective in the early period after the policy implementation (2013–2016), and the *AP* decline slows down or even rebounds in the later period of the policy. For example, Guangdong’s *AP* levels showed a significant downward trend compared to synthetic Guangdong after the policy implementation, but the *AP* levels slightly rebounded in 2017. (2) The emission reduction effect of *APPA* on *AP* varies significantly among provinces. For example, while in the same Beijing–Tianjin–Hebei region, Beijing and Hebei show huge differences in emission reduction effects. Beijing shows a significant downward trend in *AP* levels compared to synthetic Beijing. Because Beijing’s economic, political, and cultural development is superior to that of other provinces, the implementation of the policy will receive more financial and social support than other regions to promote the implementation of the policy. However, after the implementation of the policy, the decline of *AP* in Hebei and synthetic Hebei did not show a significant difference. The reason may be that although Hebei is located in the Beijing–Tianjin–Hebei region, it does not have the capital city advantage of Beijing and still maintains a production model based on a traditional industrial structure. At the same time, many problems in the policy system’s top–down implementation have led to a relatively poor *AP* reduction in Hebei. In addition, the *AP* emission reduction effect in Jiangsu, Zhejiang and Shanghai (Shanghai, Zhejiang, Jiangsu and Anhui) is better than that in Beijing, Tianjin and Hebei and the Pearl River Delta region. It may be because in the Jiangsu, Zhejiang, and Shanghai regions, compared to the other two regions, industrial transformation is completed earlier, the industrial chain is more complete, and multiple entities collaborate to promote regional ecological and environmental integration *AP* energy saving and emission reduction.

### 4.3. Robustness Tests

#### 4.3.1. DID

DID is still the dominant method for exploring policy effects today because of its effectiveness in avoiding the endogeneity of policy issues and omitted variables. We construct DID models to verify the robustness of the previous results.

(8) $$AirPollution_{it}=\alpha_{0}+\alpha_{1}treated_{it}\times time_{it}+\alpha_{2}Control+u_{i}+\lambda_{t}+\varepsilon_{it}$$

In Equation (8), the $AirPollution_{it}$ is an *AP* variable, representing *AP* in year *t* in province *i*. *treat_it_* is an individual dummy variable representing 1 for provinces in the treatment group in year *t* and 0 for other provinces. *Time_it_* is a time dummy variable bounded by the year of policy implementation (2013), with the year before policy as 0 and the other years defined as 1. *Control_it_* is a set of observable control variables with an impact on *AP* The cross-term *treat_it_ × time_it_* represents the province dummy variable after the policy implementation, and the coefficient *α_1_* represents the net effect of *APPC* on *AP*. *u_i_* and *λ_t_* represent the individual and time fixed effects, respectively, and *ε_it_* represents the random error term.

Table 6 shows the regression results for the two-way fixed effects; (1) and (2) represent the results with and without the control variables, respectively, and it can be seen that the coefficients of the interaction terms are negative and pass the significance level test, indicating that the implementation of *APPC* significantly reduces the *AP* in the priority provinces. This also proves that the baseline results are robust.

#### 4.3.2. Time to Change Policy

**Figure 4:**
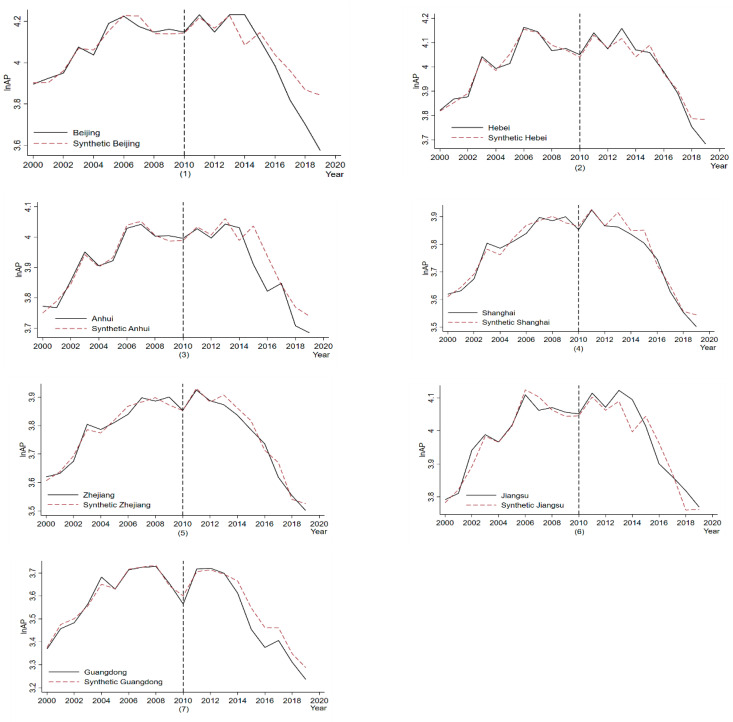
Robustness Tests for Changing Policy Point-in-Time.

We assume that the policy is advanced to 2010 and re-run the synthetic control analysis. Figure 4 shows the synthetic control results after changing the time point of the policy. It can be seen that after changing the policy time point, the trends of *AP* water in the treatment and synthetic provinces are almost identical and do not fluctuate due to the policy shock until around 2013, when the gap started to appear. It indicates that the hypothesis of using 2010 as the policy shock time point is invalid and further proves our previous results’ robustness.

#### 4.3.3. Placebo Test

**Figure 5:**
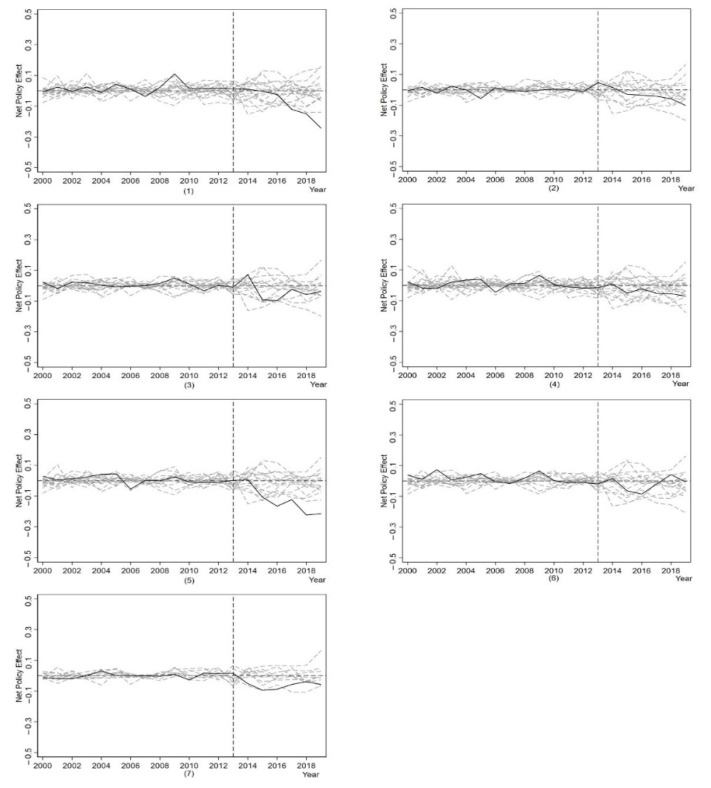
Placebo test result−2. Note: (1)–(7) represent Beijing, Hebei, Anhui, Shanghai, Zhejiang, Jiangsu, and Guangdong.

A placebo test can be used to determine whether the synthetic control method’s policy effect is statistically significant. The placebo test involves assuming all 22 provinces in the control group as the treatment group, conducting synthetic control for each province, and calculating the difference between the true and synthetic values. If the reduction in air pollution in the experimental group should be much greater than that in the control group, it indicates that *APPA* effectively reduces air pollution. It should be noted that if the difference of *AP* is larger after the policy time point, it may also be caused by the poor fitting degree before the policy time point, which is unrelated to the policy implementation. The synthetic control method requires a well-fitting synthetic control object for each province before the policy implementation. If a province has a large root of the mean square prediction error (RMSPE), then the larger predictive variable differences obtained later in the policy do not reflect the effect. Therefore, to avoid problems caused by too large fitting errors, we exclude samples with RMSPE greater than two times the experimental provinces. Table 7 shows the results of Figure 5, showing the placebo test for each province with the solid black line representing the experimental provinces and the dashed line showing the policy effects for the control group provinces. If the overall trend of the solid line is much lower than the dashed line after the policy is implemented, it indicates that the policy effect is significant. For the air pollution abatement effect, the number of dashed lines that exceed the solid line along the overall trend is divided by the total number of dashed lines (excluding the excluded samples and solid lines), which are denoted as P(Φ). Since there were only 22 samples in the control group in this study, and then after excluding the samples with too high RMSPE, the remaining samples were small, it was difficult to meet the significance condition of the statistic P < 0.05 (95% confidence level), the confidence level was relaxed appropriately. P(Φ) < 0.2 was used as the criterion for whether the placebo test was significant.

In Beijing, for example, the number of dashed lines after excluding samples is 17. After the implementation of the policy, there is no overall trend of dashed lines over the lower edge of the solid line, i.e., P(Φ) = 0/17 < 0.2. This indicates that the decrease in air pollution in Beijing is significantly greater than that of the placebo test, which is significant. However, in Hebei, for example, after excluding the sample, there are 13 dashed lines, and the overall trend of the dashed line exceeds the lower edge of the solid line in 2 cases, P(Φ) = 2/13 = 0.15 < 0.2, and the placebo test is also significant. However, P(Φ) = 4/17 = 0.235 for Shanghai; it can be concluded that *APPA* does not entirely cause the decrease in air pollution in Shanghai. The placebo test (Table 7) shows that all experimental provinces passed the placebo test except Shanghai, ensuring our conclusion’s robustness.

### 4.4. Mechanism Testing

Based on the previous results, *APPA* reduced *AP* in the treatment provinces. Many studies in the past usually concluded that there is an inverted U-shaped curve between economic development and air quality. Excessive economic growth rates in most developing countries in the early stages have led to severe *AP*. The relationship between *AP* and population size has never been conclusively established. However, many scholars believe that the increasing population in cities is accompanied by rapid growth in the service sector and technological innovation, which contribute to cleaner cities. In addition, many scholars also believe that controlling carbon emissions and optimizing industrial structure can promote air quality improvement. Therefore, we select the level of economic development, population size, carbon emission and industrial structure as mechanism variables to investigate the influence mechanism of *APPA* to reduce *AP* and construct the following model.

(9) $$Mechanism_{it}=\alpha_{0}+\alpha_{1}treat*time+u_{i}+\lambda_{t}+\varepsilon_{it}$$

In Equation (9), *Mechanism_it_* represents the impact mechanism variables (*lnECO, lnPOP, lnCO_2_, lnIS*). *treat_it_* is the province dummy variable, *time_it_* is the policy time dummy variable, *u_i_* and *λ_t_* represent the province individual effect and time effect, and *ε_it_* is the random perturbation term.

The results in Table 8 show that all interaction coefficients pass the significance level test. *lnECO* has a coefficient of −0.1938, indicating that the per capita GDP of the treatment province decreased after *APPA*. The reason may be that the treatment province was pressured by environmental policies to improve air quality by slowing down its economic development and shifting to high-quality development. *lnPOP* has a coefficient of 0.0853, indicating that *APPA* expanded the population size of the treatment province, suggesting that *APPA* reduced *AP* through population clustering. The coefficients of *lnCO_2_* and *lnIS* are −0.1912 and 0.0907, indicating that both carbon emissions and the share of secondary industry in the treatment provinces have decreased, suggesting that controlling carbon emissions and optimizing industrial structure are important channels to improve air quality. The absolute values of the coefficients of *lnECO* and *lnCO*_2_ are significantly higher than those of *lnPOP* and *lnIS*, indicating that promoting high-quality economic development and controlling carbon emissions have a more important impact on reducing *AP*.

## 5. Conclusions and Policy Implication

### 5.1. Conclusions

This study explored the effect of *APPA* on *AP* using a synthetic control method based on panel data from 30 provinces for 2000 to 2019. This paper draws the following conclusions:

(1) After *APPA*, the treatment provinces all showed a more significant reduction in *AP* compared with synthetic provinces. It indicates that *APPA* significantly reduces *AP* in the treatment provinces, and subsequent robustness tests verify the baseline results. Beijing and Zhejiang have significantly better air pollution reduction effects than other provinces. However, *APPA* produces less persistent policy effects in some provinces (i.e., Anhui and Jiangsu); the rate of *AP* decline slows down or even rebounds in the later stages of the policy.
(2) The emission reduction effect of *APPA* on *AP* varies significantly both among provinces in the same region and among different regions. For example, Beijing and Hebei belong to the same Beijing–Tianjin–Hebei region, but the emission reduction effect greatly differs. In addition, the *AP* abatement effect in the Yangtze River Delta is better than that in the Beijing–Tianjin–Hebei and Pearl River Delta regions.
(3) The results of the mechanism test show that *APPA* reduces *AP* through high quality economic development, population agglomeration, control of carbon emissions and optimization of energy mix. The effects are in the order of strongest to weakest (*ECO, POP, CO_2_, IS*).

### 5.2. Policy Implication

Although *APPA* has significantly contributed to reducing *AP*, it is even more important to introduce effective long-term policies to combat *AP*. Therefore, this study makes the following policy recommendations.

(1) The formulation of government environmental policies should consider the regional resource endowment, economic development level and other conditions and make policies that meet the region’s characteristics according to local conditions. For example, the Beijing–Tianjin–Hebei region’s main task is to eliminate low-end, polluting industries and achieve industrial transfer to reduce environmental pressure. The Yangtze River Delta and the Pearl River Delta have already reduced emissions and promoted synergistic regional environmental and economic development while achieving industrial clustering.
(2) This study’s findings show significant differences in the effects of *AP* management in different provinces. *AP* management is a long-term process, and if we want to tackle *AP* at its root, we must adhere to the approach of regional coordination and joint prevention and control and strengthen policy communication and coordination to win the blue sky defense war.
(3) The results of the influence mechanism test show that high-quality economic development, population agglomeration, control of carbon emissions and optimization of energy structure are all important channels to reduce *AP*. Therefore, we need to change and transform the economic development mode, develop the circular economy, optimize the industrial structure and promote industrial transformation and upgrading. For example, we could promote the Beijing–Tianjin–Hebei region and other heavily polluted areas to revise the access conditions for high energy consumption, high pollution and resource-based industries, and clarify indicators such as resource and energy conservation and pollutant emissions. In addition, we could also promote population urbanization and create clean, low-carbon cities scientifically and rationally.

However, this paper also has some limitations. We will obtain a more nuanced conclusion when the quantitative results of the *APPA* policy effects are combined. In addition, the impact of the pandemic should be considered for inclusion in the research framework after COVID-19 in future studies.

## Figures and Tables

**Table 1:** Energy Conversion Factor.

Energy Type	Carbon Dioxide
Raw Coal	1.9003
Coke	2.8604
Crude Oil	3.0202
Gasoline	2.9251
Kerosene	3.0179
Diesel	3.0959
Fuel Oil	3.175
Liquefied Petroleum Gas	3.1013
Natural Gas	21.622

**Table 2:** Variable Measurements and Sources.

Variables	Measurement	Unit	Source
*AP*	Concentration of PM_2.5_	μg/m^3^	Atmospheric Composition Analysis Organization
*ECO*	GDP per capita	Yuan/person	China Statistical Yearbook
*POP*	Population size	10,000 people	China Statistical Yearbook
*CO*_2_	Save CO_2_ emissions	million tons	China Statistical Yearbook
*IS*	Secondary Industry	%	China Statistical Yearbook

**Table 3:** Descriptive Statistics.

Group	Variable	N	Mean	sd	Min	Max
Treatment group	*PM_2.5_*	140	47.7	10.54	25.44	70.44
	*ECO*	140	51,702.51	35,611.66	4867.41	164,220
	*POP*	140	5706.75	2774.2	1357	11,521
	*CO_2_*	140	40,904.93	24,749.03	10,049.15	94,794.99
	*IS*	140	44.8	9.78	16.2	56.6
Control group	*PM_2.5_*	460	38.54	14.21	9.57	85.63
	*ECO*	460	29,549.84	22,075.69	2661.56	120,711
	*POP*	460	4024.52	2493.34	517	10,070
	*CO_2_*	460	29,999.29	26,095.49	547.5379	151,523.5
	*IS*	460	45.75	7.57	19.76	61.5
Total	*PM_2.5_*	600	40.68	13.98	9.57	85.63
	*ECO*	600	34,718.79	27,489.22	2661.56	164,220
	*POP*	600	4417.04	2656.46	517	11,521
	*CO_2_*	600	32,543.94	26,177.52	547.5379	151,523.5
	*IS*	600	45.53	8.14	16.2	61.5

**Table 4:** Comparison of predictor variables.

	*lnECO*	*lnPOP*	*lnIS*	*lnCO_2_*	*lnAP* (2000)	*lnAP* (2006)	*lnAP* (2010)
Beijing	10.7659	7.3948	3.3710	9.3905	3.8960	4.1900	4.1478
Synthetic Beijing	9.5898	9.0916	3.9581	10.6459	3.8959	4.2145	4.1454
Hebei	9.7250	8.8436	3.9509	10.9322	3.8212	4.1632	4.0702
Synthetic Hebei	9.7629	8.8452	3.9633	10.7669	3.8189	4.1480	4.0542
Anhui	9.2911	8.7289	3.8353	10.0145	3.7721	4.0292	3.9961
Synthetic Anhui	9.8079	8.6492	3.9319	10.6340	3.7368	4.0411	3.9922
Shanghai	10.96703	7.5421	3.8199	10.0236	3.6197	3.8397	3.8033
Synthetic Shanghai	9.896227	8.6023	3.9335	10.4393	3.5990	3.8897	3.8033
Zhejiang	10.3239	8.5148	3.9588	10.3069	3.5501	3.6719	3.6940
Synthetic Zhejiang	9.7799	8.1752	3.8927	10.1261	3.5296	3.7280	3.7052
Jiangsu	10.2488	8.9351	3.9827	10.7251	3.7918	4.0997	4.0193
Synthetic Jiangsu	9.8629	8.6790	3.9690	10.8428	3.7714	4.1156	4.0287
Guangdong	10.2014	9.1093	3.9303	10.6028	3.3686	3.7138	3.5646
Synthetic Guangdong	9.6877	8.3934	3.7995	9.5839	3.3749	3.7130	3.5881

**Table 5:** Synthetic province weighting factor.

Synthetic Province	Synthetic Weight						
Synthetic Beijing	Henan	Hubei	Ningxia	Shandong			
	0.565	0.059	0.044	0.332			
Synthetic Hebei	Henan	Liaoning	Shandong	Shanxi	Xinjiang		
	0.355	0.129	0.359	0.075	0.082		
Synthetic Anhui	Henan	Inner Mongolia	Shandong	Xinjiang			
	0.190	0.125	0.476	0.208			
Synthetic Shanghai	Fujian	Henan	Heilongjiang	Liaoning	Inner Mongolia	Shandong	Xinjiang
	0.025	0.165	0.021	0. 048	0.120	0.342	0.054
Synthetic Zhejiang	Fujian	Henan	Heilongjiang	Liaoning	Inner Mongolia	Shandong	Xinjiang
	0.162	0.072	0.154	0.083	0.030	0.130	0.368
Synthetic Jiangsu	Inner Mongolia	Ningxia	Shandong	Shanxi	Xinjiang		
	0.044	0.024	0.655	0.182	0.094		
Synthetic Guangdong	Fujian	Guangxi	Hainan	Shandong			
	0.406	0.430	0.028	0.136			

**Table 6:** DID results.

VARIABLES	(1)	(2)
	*lnAP*	*lnAP*
*Treat* × *time*	−0.1305 ***	−0.1254 ***
	(0.0185)	(0.0195)
*lnECO*		−0.1180 ***
		(0.0385)
*lnPOP*		−0.3116 ***
		(0.0781)
*lnCO_2_*		0.0681 ***
		(0.0239)
*lnIS*		−0.1299 **
		(0.0548)
Constant	3.5193 ***	6.9513 ***
	(0.0167)	(0.8062)
Observations	600	600
R-squared	0.654	0.673
Number of code	30	30
City FE	Yes	Yes
Year FE	Yes	Yes
Control variables	No	Yes

Note: Standard errors in parentheses. *** *p* < 0.01,** *p* < 0.05.

**Table 7:** Placebo test result-1.

Province	RMSPE	Number of Dashed Lines	Number of Edge Dashes	P(Φ)	Placebo Test
Beijing	0.020381	17	0	0	Significant
Hebei	0.015512	13	2	0.154	Significant
Anhui	0.015186	14	2	0.1430	Significant
Shanghai	0.020581	17	4	0.235	Insignificant
Zhejiang	0.023825	15	0	0	Significant
Jiangsu	0.024262	16	3	0.1875	Significant
Guangdong	0.014185	12	1	0.083	Significant

**Table 8:** Regression results of Mechanism Testing.

VARIABLES	(1)	(2)	(3)	(4)
	*lnECO*	*lnPOP*	*lnCO_2_*	*lnIS*
*Treat* × *time*	−0.1938 ***	0.0853 ***	−0.1912 ***	−0.0907 ***
	(0.0269)	(0.0113)	(0.0363)	(0.0192)
Constant	8.8928 ***	8.0952 ***	9.2828 ***	3.7780 ***
	(0.0243)	(0.0102)	(0.0327)	(0.0174)
Observations	600	600	599	600
R-squared	0.970	0.427	0.846	0.446
Number of provinces	30	30	30	30
City FE	Yes	Yes	Yes	Yes
Year FE	Yes	Yes	Yes	Yes
Control variables	Yes	Yes	Yes	Yes

Note: Standard errors in parentheses. *** *p* < 0.01.

## Data Availability

PM_2.5_ data were obtained from the Atmospheric Composition Analysis Organization. The predictor variable data were obtained from each province’s China Statistical Yearbook and Statistical Yearbooks.
